# Supplementary material for: Spatial summation of pain is associated with pain expectations: Results from a home-based paradigm
Source: PLoS One. 2024 Feb 1;19(2):e0297067. doi: 10.1371/journal.pone.0297067 (PMC10833545; doi:10.1371/journal.pone.0297067)
Supplement: S1 Text — (DOCX) [file pone.0297067.s001.docx]

**S2 Text. Protocol deviations**. The following deviations from the pre-registered protocol must be acknowledged: i) the declared sample of N=90 was not reached due to rigorous inclusion criteria and the peak of incidence rates of COVID-19 during recruitment for this experiment, ii) correlations are reported based on Spearman`s rank correlation coefficient, iii) apart from polynomial contrast results, pairwise comparisons are reported, iv) neither outcomes nor residuals were normally distributed so General Linear Model was replaced by General Estimated Equations, however both analyses provided similar results.
