# Supplementary material for: Spatial summation of pain is associated with pain expectations: Results from a home-based paradigm
Source: PLoS One. 2024 Feb 1;19(2):e0297067. doi: 10.1371/journal.pone.0297067 (PMC10833545; doi:10.1371/journal.pone.0297067)
Supplement: S2 Text — (DOCX) [file pone.0297067.s002.docx]

**S3 Text. Exclusion criteria.** Specific exclusion criteria: Raynaud's phenomenon (cold fading of the fingers, followed by blush numbness and redness), cold allergy (cold urticaria), cold intolerance, cryoglobulinemia, paroxysmal cold hemoglobinuria, rheumatic diseases (e.g. osteoarthritis, rheumatoid arthritis, fibromyalgia, systemic lupus erythematosus, etc.), pheochromocytoma, skin sensitivity disorders, sympathetic neuropathies, cardiovascular diseases (coronary artery disease, chronic heart failure, cardiac insufficiency), neuropathy (e.g. cardiovascular disorders), adrenal pheochromocytoma, sensory skin disorders, sympathetic neuropathies, cardiovascular diseases (coronary artery disease, chronic heart failure class III and IV according to NYHA), hypothyroidism, purulent gangrenous skin lesions, local blood flow disorders.
