# Supplementary figures and images for: Spatial summation of pain is associated with pain expectations: Results from a home-based paradigm
Source: PLoS One. 2024 Feb 1;19(2):e0297067. doi: 10.1371/journal.pone.0297067 (PMC10833545; doi:10.1371/journal.pone.0297067)

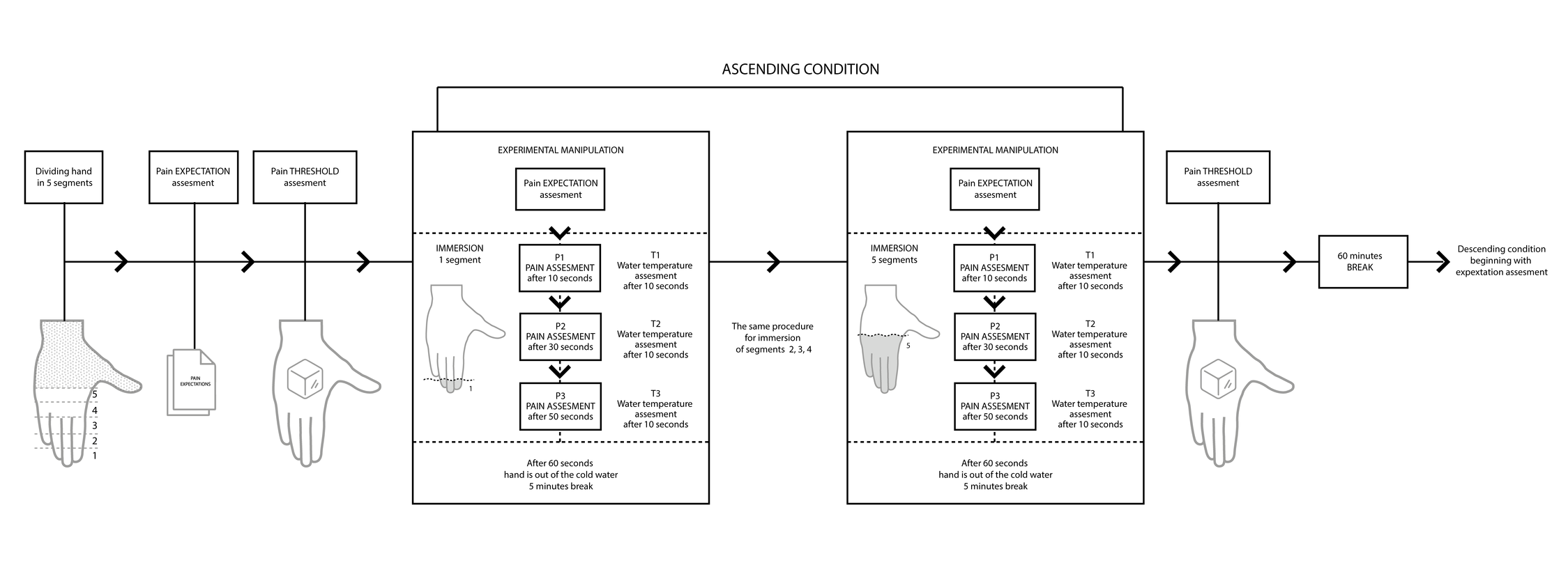

Supplement: S1 Fig — Study procedures. In each condition single trial lasted 60 seconds (regardless of the number of segments involved). Each trial started with a question about expected pain intensity. Before and after each experimental condition cold pain thresholds (PTCOLD) were tested on the examined limb. Participants were instructed to immerse their hand up to the line which separated a given number of segments. Participants were prompted to rate their pain intensity on the VAS scale at the following time points: after 10, 30 and 50s. Inter-trial intervals were set at 5 minutes. The interval between each condition was one hour. (TIF) [file pone.0297067.s003.tif]
