# Supplementary material for: Spatial summation of pain is associated with pain expectations: Results from a home-based paradigm
Source: PLoS One. 2024 Feb 1;19(2):e0297067. doi: 10.1371/journal.pone.0297067 (PMC10833545; doi:10.1371/journal.pone.0297067)
Supplement: S1 Table — SD- standard deviations. (DOCX) [file pone.0297067.s004.docx]

**S1 Table. Means and standard deviations for temperatures in pilot study (training)**

| **Time of measurement** | **Segment** | **Ascending (SD)** | **Descending (SD)** |
| --- | --- | --- | --- |
| 10s | 1 | 5.28°C (0.44) | 4.91°C (0.51) |
| 30s | 1 | 5.30°C (0.43) | 4.94°C (0.48) |
| 50s | 1 | 5.33°C (0.40) | 4.91°C (0.51) |
| 10s | 2 | 5.22°C (0.35) | 5.10°C (0.46) |
| 30s | 2 | 5.20°C (0.40) | 5.08°C (0.53) |
| 50s | 2 | 5.24°C (0.41) | 5.14°C (0.43) |
| 10s | 3 | 5.10°C (0.26) | 5.05°C (0.26) |
| 30s | 3 | 5.05°C (0.35) | 5.05°C (0.27) |
| 50s | 3 | 5.04°C (0.32) | 5.08°C (0.25) |
| 10s | 4 | 5.13°C (0.25) | 5.16°C (0.24) |
| 30s | 4 | 5.11°C (0.35) | 5.00°C (0.46) |
| 50s | 4 | 5.01°C (0.36) | 4.94°C (0.50) |
| 10s | 5 | 5.12°C (0.37) | 5.13°C (0.15) |
| 30s | 5 | 4.93°C (0.41) | 5.04°C (0.19) |
| 50s | 5 | 4.89°C (0.43) | 5.03°C (0.27) |
