# Supplementary material for: Spatial summation of pain is associated with pain expectations: Results from a home-based paradigm
Source: PLoS One. 2024 Feb 1;19(2):e0297067. doi: 10.1371/journal.pone.0297067 (PMC10833545; doi:10.1371/journal.pone.0297067)
Supplement: S2 Table — SD- standard deviations. (DOCX) [file pone.0297067.s005.docx]

**S5 Table. Measurement of pain thresholds**

| **Pain threshold (seconds)** | **Mean (SD)** |
| --- | --- |
| Before Ascending | 73.28 (134.88) |
| After Ascending | 76.53 (125.87) |
| Before Descending | 74.10 (139.38) |
| After Descending | 55.73 (85.08) |
