# Supplementary material for: Spatial summation of pain is associated with pain expectations: Results from a home-based paradigm
Source: PLoS One. 2024 Feb 1;19(2):e0297067. doi: 10.1371/journal.pone.0297067 (PMC10833545; doi:10.1371/journal.pone.0297067)
Supplement: S3 Table — 1/5 –Segment 1, 2/5 –Segments 1 to 2, 3/5- Segments 1 to 3, 4/5 –Segments 1 to 4, 5/5- Segments 1 to 5, SD, standard deviations. (DOCX) [file pone.0297067.s006.docx]

**S6 Table. Means and standard deviations for pain intensity reported during cold water immersions**

| **Condition** | **Time of measurement** | **Segment(s)** | | | | |
| --- | --- | --- | --- | --- | --- | --- |
|  |  | **1/5** | **2/5** | **3/5** | **4/5** | **5/5** |
|  |  | **Mean (SD)** | **Mean (SD)** | **Mean (SD)** | **Mean (SD)** | **Mean (SD)** |
| **Ascending** | **10 s** | 8.54 (11.31) | 10.60 (12.27) | 13.85 (15.18) | 17.44 (17.45) | 22.77 (20.39) |
|  | **30 s** | 13.82 (15.42) | 16.23 (16.39) | 21.13 (20.53) | 27.97 (23.35) | 34.89 (25.43) |
|  | **50 s** | 18.92 (20.09) | 23.07 (20.40) | 30.63 (25.09) | 36.57 (26.53) | 43.19 (29.24) |
| **Descending** | **10 s** | 6.17 (11.49) | 10.64 (14.35) | 14.30 (14.59) | 20.42 (18.63) | 23.48 (21.95) |
|  | **30 s** | 8.50 (13.32) | 13.61 (17.60) | 20.45 (19.84) | 29.73 (24.07) | 36.50 (25.62) |
|  | **50 s** | 10.79 (15.44) | 17.19 (20.62) | 35.63 (23.16) | 37.80 (25.56) | 47.73 (29.10) |
