# Supplementary material for: Spatial summation of pain is associated with pain expectations: Results from a home-based paradigm
Source: PLoS One. 2024 Feb 1;19(2):e0297067. doi: 10.1371/journal.pone.0297067 (PMC10833545; doi:10.1371/journal.pone.0297067)
Supplement: S4 Table — 10s - pain intensity measured after 10 seconds, 30s - after 30 seconds, 50s - after 50 seconds of immersion, Mean ‐ mean pain intensity from each immersion, B ‐ unstandardized coefficients, SE ‐ standard error, β ‐ standardized coefficients, p–significance value. (DOCX) [file pone.0297067.s007.docx]

**S7 Table. Slopes and intercepts for relationships between pain and number of stimulated segments**

|  |  | **Ascending** | | | |  | **Descending** | | | |
| --- | --- | --- | --- | --- | --- | --- | --- | --- | --- | --- |
| **Variables** | **R^2^** | **B** | **SE** | **β** | ***p*** | **R^2^** | **B** | **SE** | **β** | ***p*** |
| 10s | 0.97 | 3.53 | 0.34 | 0.98 | < 0.01 | 0.99 | 4.44 | 0.23 | 0.99 | < 0.001 |
| 30s | 0.96 | 5.38 | 0.55 | 0.98 | < 0.01 | 0.99 | 7.21 | 0.39 | 0.99 | < 0.001 |
| 50s | 0.99 | 6.20 | 0.27 | 0.99 | < 0.001 | 0.98 | 9.45 | 0.60 | 0.99 | < 0.001 |
| Mean | 0.98 | 5.08 | 0.38 | 0.99 | < 0.001 | 0.99 | 7.06 | 0.39 | 0.99 | < 0.001 |
