# Supplementary material for: Spatial summation of pain is associated with pain expectations: Results from a home-based paradigm
Source: PLoS One. 2024 Feb 1;19(2):e0297067. doi: 10.1371/journal.pone.0297067 (PMC10833545; doi:10.1371/journal.pone.0297067)
Supplement: S5 Table — 1/5 –Segment 1, 2/5 –Segments 1 to 2, 3/5- Segments 1 to 3, 4/5 –Segments 1 to 4, 5/5- Segments 1 to 5, SD, standard deviations. M, median. IQR, interquartile range. (DOCX) [file pone.0297067.s008.docx]

**S8 Table. Pain-related expectations measured prior to cold water immersions**

|  | **Ascending** | | | | **Descending** | | | |
| --- | --- | --- | --- | --- | --- | --- | --- | --- |
| **Segment** | **Mean** | **SD** | **M** | **IQR** | **Mean** | **SD** | **M** | **IQR** |
| 1/5 | 20.77 | 21.91 | 14 | 29 | 22.45 | 21.44 | 16.5 | 29.5 |
| 2/5 | 23.58 | 19.99 | 15 | 29 | 25.70 | 19.38 | 23.5 | 25.5 |
| 3/5 | 25.85 | 19.70 | 21 | 29 | 29.67 | 19.23 | 28 | 28.5 |
| 4/5 | 34.74 | 23.60 | 31 | 37 | 34.27 | 21.09 | 33 | 32 |
| 5/5 | 42.04 | 27.11 | 42 | 51 | 37.05 | 23.58 | 36.5 | 40 |
