# Supplementary material for: Spatial summation of pain is associated with pain expectations: Results from a home-based paradigm
Source: PLoS One. 2024 Feb 1;19(2):e0297067. doi: 10.1371/journal.pone.0297067 (PMC10833545; doi:10.1371/journal.pone.0297067)
Supplement: S6 Table — (DOCX) [file pone.0297067.s009.docx]

**S9 Table. The number of participants tested by each examiner**

| **Examiner** | **Number of assessed participants** | **Percent** |
| --- | --- | --- |
| E1 | 4 | 5.882 |
| E2 | 4 | 5.882 |
| E3 | 3 | 4.412 |
| E4 | 6 | 8.824 |
| E5 | 7 | 10.294 |
| E6 | 9 | 13.235 |
| E7 | 12 | 17.647 |
| E8 | 5 | 7.353 |
| E9 | 2 | 2.941 |
| E10 | 6 | 8.824 |
| E11 | 2 | 2.941 |
| E12 | 1 | 1.471 |
| E13 | 7 | 10.294 |
