# Supplementary material for: Spatial summation of pain is associated with pain expectations: Results from a home-based paradigm
Source: PLoS One. 2024 Feb 1;19(2):e0297067. doi: 10.1371/journal.pone.0297067 (PMC10833545; doi:10.1371/journal.pone.0297067)
Supplement: Attachment — Submitted filename: HOME_responseToreviewers_20231207.docx [file pone.0297067.s011.docx]

**Reviewer #1**

The manuscript as it is presented now seems to be much more clear and the readability is improved a lot. Moreover, the message (and evidence) of conducting these experiments outside the lab (but with strict procedures and reporting of possible influential factors) is conveyed much better and will hopefully help the academic world to progress/translate into the clinic, give opportunities to researches without such a lab and hopefully strengthen our understanding of pain further to help our patients in pain.

**Response:** Thank you very much for your positive feedback and suggestions which improved our manuscript.

As a minor last remark, COVID-19 as keyword doesn't seem appropriate in my opinion. Possibly the editorial office can erase it, since the study doesn't study COVID, and we preferably don't want to give 'noise' to systematic review searchers on the subject.

**Response:** Thank you for remark. We removed “COVID-19” as a keyword.

Finally, I want to thank the authors for their great work!

**Response:** Thank you so much for your time to review our manuscript!

**Reviewer #2**

I thank the Authors for their responses to my comments. I think the manuscript as greatly improved since the previous stage and is almost ready for publication. I however still have a few remarks, exposed hereunder.

**Response:** We are very thankful for your positive feedback and further set of comments on our study

Main points:
-The Authors state that “Spatial summation of pain (SSp) may be characterized as an increase of pain intensity when [… the] distance [4,5] between stimulated areas are enlarged [3].” The distance between stimulated areas has been shown to modulate spatial summation, something referred to as “lateral inhibition” by certain authors, but this is not spatial summation proper, a concept with a long history which has always been about larger stimulation surface area. I would therefore invite the Authors to rephrase this sentence. Changing the definition of spatial summation may put the pain literature at odds with the haptics and thermosensation literature.

**Response:** Thank you for your feedback. We rephrased part of introduction referring to definition of spatial summation and spatial conditions under which it occurs:

*“Spatial summation of pain (SSp) is characterized by an increase of perceived pain intensity when the area of nociception is enlarged [1–3]. Furthermore, pain thresholds decrease as the area of noxious stimulation increases [4–6] which can also be seen as a spatial summation manifestation. There are also studies indicating that SSp can occur when the area of nociception is not contiguous [4,5]. However, studies show that when the separation between stimulated areas exceeds 30 cm (in case of cold pain) [7] or 10 cm (in the case of heat pain) [8], SSp no longer occurs”*

-“An interesting observation is that there is no exponential or linear increase in pain intensity during a linear increase in the stimulated body area [1] or in the distance between the stimuli [1,4]. Instead, an increase in pain seems to be characterized by a relatively quick saturation when the area of the body exposed to noxious stimulation is expanded in a linear fashion, further suggesting that inhibitory processes are involved in SSp.” I am a bit confused by these statements as the classically described stimulus-response function in psychophysics is concave-down increasing, which led Fechner to propose a logarithmic psychophysical law in the 19th century. Stevens later showed that Fechner was wrong and that a power law should be used instead but this only marginally affects the shape of the function, not the orientation of the concavity. The observations of the Authors are therefore perfectly in line with the psychophysical literature but the introduction seems to suggest otherwise. Additionally, this type of behavior could be completely accounted for by partially overlapping receptive fields at the different levels of the neural hierarchy and I therefore think that the Authors should be more cautious when designating inhibitory mechanisms as a cause for this phenomenon.

**Response:** Thank you for this valid point. We would like to clarify the meaning of this cited sentence. We do not refer to sensory experience magnitude which correspond with stimulus intensity (magnitude), but to experience magnitude corresponding to the “size” of the stimulated area. What is interesting in these early psychophysical studies is that percept increased exponentially (positive exponent). However, pain seems to rather increase logarithmically when the stimulus intensity is constant, but the area increases linearly – this is what we were referring to. Along the same lines, percept seems to be more linear when the area or “size” of the stimulation increases more exponentially, like in the current study. We changed the language in the following part of the introduction:

*“**An interesting observation is that there is no exponential increase in pain intensity during a linear increase in the “size” of the stimulated area [1],* *a typical stimulus-response pattern when the stimulus are (size) is constant but the noxious intensity increases linearly [13,14].”*

-I appreciate the fact that the Authors enthusiastically adopted my suggestion of temporal summation as a mechanism explaining the difference in spatial summation between the time points but I think a more cautious style should be adopted when mentioning it as it is only a putative mechanism that could account for part of the effect. On the other hand, I think more emphasis should be put on the fact that, due to the slowness of heat conduction through the skin, from the point of view of the primary afferent (i.e. at the depth of the receptors), the temperature must have been quite different at the different time-points.

**Response:** Thank you for your feedback. We rephrased some parts of the manuscript related to temporal summation:

Abstract: *“**This study suggests that SSp is associated with pain expectations and can be formed by a mixture of excitatory and inhibitory mechanisms* ***potentially*** *driven by temporal characteristics of neural excitation”*

Discussion: *“**Moreover, SSp can be* ***potentially*** *driven by temporal characteristics of neural excitation”.*

We also rephrased part of the discussion to emphasize role of temperature:

Physiological mechanisms of SSp:

*“The curve representing the summation trajectory was steeper when the last measurements were considered (50s). During the immersion in cold water, the hand temperature had to change with time, hence, observed SSp could be driven by the gradual activation of deeply located primary afferents. This may indicate that SSp can be partially mediated by temporal and/or spatial summation of nociceptors innervating deeper layers of soft tissues [54] and possibly deep veins [55]”.*

-“In our study, this interaction was replicated, although it was prominent when only the immersion of the first segment (fingertips) was compared.” I think that a more nuanced approach is needed here. In the study of Marchand and Arsenault, almost no spatial summation was observed in the increasing condition whereas a very clear pattern of spatial summation was observed in the decreasing condition. In the case of the present study, pain ratings are largely colinear with an effect similar to that reported by Marchand and Arsenault but of much smaller magnitude only apparent at the 50 s time point. I would say that the replication was partially successful, which does not reduce the value of the Author’s study which nicely shows that this effect seems there but is a bit more subtle than what could have been expected from the Marchand and Arsenault paper. This discrepancy may come from the different type of stimulus (noxious cold vs noxious heat) something that could be discussed (heat pain tends to arise faster than cold pain, which could maybe explain why the effect seemed present only at the later time points).
Off note, when looking at the “slope” of the rating-surface relationship for time point 50s, there seems to be an overall reduction of spatial summation for the ascending condition (flatter slope), something that is maybe not entirely capture by the current data analysis (as different surfaces are modelled as factors) but goes in the direction of the Authors hypothesis.

**Response:** Thank you for this valid point. We extended the discussion section on differences between our and Marchand & Arsenault (2002) study. We also calculated regression coefficients in order to directly compare the findings to result from 2002 (especially, slopes and intercepts). We added following changes:

Data extraction and statistical analysis:

*“Linear regression was calculated to describe the relationship between pain and number of stimulated segments – similarly to the described procedures in the Marchand & Arsenault study [3]”*

Results:

*“ (…) and regressions coefficients:* *Slope of pain increase was steeper in descending compared to ascending condition, however, the most noticeable difference in steepness was found for pain measured at 50s (B = 9.45 vs. 6.20, see Table S9)”*

We also emphasized some key differences between the current study and experiment from 2002:

Discussion:

*“These differences include the localization of noxious stimulation as well as the overall size of the stimulated surface area. Only the hand was used for the current study and was divided into 5 segments, while Marchand & Arsenault [3] used the entire arm and divided it into 8 segments. Secondly, the type of noxious stimulation was different. Here, noxious cold stimulation was used, compared to noxious heat stimulation in the previous report. The type of noxious stimuli could explain the results as the most noticeable difference of pain increase between the two conditions was observed after 50s of immersion. This could be a result of temporal summation of noxious cold pain, which arises slower compared to noxious heat pain [52]. Thirdly, the study was conducted with home-based equipment outside of the laboratory.”*

Minor points:
Abstract:
“The influence of psychological factors, such as the volunteer’s expectations of pain intensity, on the actual perception of pain were also measured on a VAS.” For the reasons explained during the previous round, I think this sentence is misleading as the Author’s showed a relationship/correlation between predicted and perceived intensity but not an influence of the former on the later. I therefore recommend rephrasing the sentence accordingly.

**Response:** Thank you for pointing this out. Sentence in abstract was rephrased to:

*“**Psychological factors, such as the participants’ expectations of pain intensity were also measured on a VAS.”*

Introduction:
-“The low temperature activates nociceptive fibers [18,22,23] through low temperature sensitive ion channels TRPM8 [24] and TRPA1 [25] leading to increasing pain of mild to moderate intensity [26].” As I mentioned in my previous comments, TRPA1 may be a transducer of noxious cold but this is uncertain. I think this uncertainty should be reflected in the phrasing, e.g. “probably through low temperature sensitive ion channels TRPM8 [24] and/or TRPA1”.

**Response:** Thank you for your feedback. Word “probably” was added to the sentence.

-“Although this method does not allow adequate standardization when there are anthropometric differences, it is easy and commonly used and has been successfully employed in pain studies.” I think the Authors are too hard on themselves, standardization is not perfect, but I think it is adequate enough.

**Response:** Thank you for this comment. Sentence was rephrased to:

*“**Although standardization in this method is difficult due to e.g., anthropometric differences, it is easy and commonly used and has been successfully employed in pain experiments”*

- “Both publication bias, based on phenomenon of higher probability of publishing statistically significant findings than nonsignificant findings [33], and lack of replication studies are affecting the validity of scientific research [34]. This problem was particularly addressed by researchers in the field of psychology, leading to “crisis of confidence” in previous findings [35], but it is also found in other fields like medicine [36], economy, [37] or genetic research [38].” It is up to the Authors to decide but I don’t personally think that it is necessary to introduce the reproducibility crisis to justify an attempt at replication.

**Response:** We fully agree that there is no need to mention replication crisis to justify the replication attempt. However, still there is a negative bias towards conducting and publishing replication studies in many scientific fields. So, we decided to include this part in our paper, however, we shorten it significantly.

Methods:
-“Length and width of each segment was measured using flexible measuring tape before the main phase of experiment.” Thanks for adding this information. I am however still not sure of how these measures were transformed into surface areas. Did the Authors model the segments as rectangular parallelepipeds?

**Response:** Yes, however based on 2 dimensions as this was the easiest and the fastest approximation. During the immersion, the participants were instructed to keep their hands straight. In such position shape of the hand is similar to a parallelogram tapering slightly toward the tips of the fingers. We collected measurements of two dimensions of each segment: length (a) and width (b). Then these values were multiplied to obtain an approximated surface area for each segment (a_x_ × b_x_). Following sentence was added to the manuscript:

*“To calculate the “area” of immersed segments, surface areas (ax × bx) of each immersed segment were summed. For example: the surface area of 5/5 segments was calculated as: first segment (a_1_ × b_1_) + second segment (a_2_ × b_2_) + third segment (a_3_ × b_3_) + fourth (a_4_ × b_4_) fifth segment (a_5_ × b_5_).”*

-“The comparison between water temperature and PPTs before and after the experiment was conducted using paired Student t tests” I don’t think the PPT abbreviation is defined.

**Response:** Thank you very much for pointing this out. PPT was incorrect word, we meant *PT_COLD._* We clearly missed it. Sentence is now rephrased to:

*“The comparison between water temperature and PT_COLD_ before and after the experiment was conducted using paired Student t tests.”*

Discussion:
-“Inhibitory processes could be inferred from an interaction between the sequence and the stimulated body area (segments).” By sequence, do the Authors mean ascending/descending?

**Response:** Thank you very much for pointing this out. Correct, what we meant was a sequence of particular immersions. Sentence is now rephrased to: *“Inhibitory processes could be inferred from an interaction between the ascending and descending immersion and the stimulated body area (segments).”*

-This sentence seems to be missing a verb. “Moreover, a study on rats which showed that an increase in stimulation area from 1.9 to 18cm2 gradually decreased the frequency of convergent neuron discharges in intact, yet not spinally transected animals [52].”

**Response:** Thank you for this comment. Sentence was corrected to: *“Moreover, one study conducted on rats showed that an increase in stimulation area from 1.cm^2^ 9 to 18cm^2^ gradually decreased the frequency of convergent neuron discharges in intact, yet not spinally transected animals”*

**Reviewer #3**

I thank the authors for having integrated the manuscript with the given feedback. In my opinion, the manuscript has increased its quality and clarity. Below are some minor revisions. A general comment is that the authors could give a last round of revision, checking the grammar and the language clarity.

**Response:** Thank you very much for your feedback and for taking time to review our manuscript.

Line 70. Typo. ‘To address this IS in SSp’. The authors state one of their aims in this sentence. However, this is not so clear. I suggest stating this aim more clearly (e.g., To address this in SSp, we aim to replicate this effect, adopting the methodology from the study of Marchand & Arsenault, in the present study’.

**Response:** Thank you for pointing this out. Sentence was rephrased to: *“**To address this for the topic of SSp, one of the aims for the current study was to replicate the SSp effect, adopting the methodology from the study published by Marchand & Arsenault [3].”*

Line 78. ‘Furthermore, the current experiment…’ I would not put this in a new paragraph. I would continue in the same paragraph so that the two aims are clearly stated in one paragraph.

**Response:** Thank you for your feedback. Sentence is now in the same paragraph.

Spelling mistakes in S4 ‘after 60 seconds hands is of the cold water 5 minutes breakv’

**Response:** Thank you for this comment. S4 was corrected.

Line 443. ‘Another is that’ Paraphs missing a word ‘Another limitation is that’

**Response:** Thank you for your feedback. Word “limitation” was added to the sentence.

452. ‘Further research should focus on the manipulation of expectations to see if the subjects' level of expectations affects SSp and is able to modulate it.’ Good observation. I suggest making this comment more explicit saying that the present study is limited to the associative level (as indicated by correlations), while modulating expectations could give insights on the causal relationship between expectancy and SSp.

**Response:** Thank you very much for your suggestions. This comment was rephrased to:

*“**The present study is limited to making inferences at the association level, rather than establishing causation. Further research should focus on experimentally manipulated expectations to test if subjects' expectations affect SSp. This could give an insight on the causal relationship between expectancy and SSp”.*

459-460. I suggest writing a separate sentence for the comment on physiological measurements.

**Response:** Thank you for this comment. Separate sentence on physiological measurements was added:

“*Additionally, the inclusion of physiological measures could prove beneficial in future studies*.”

**References**

1. Adamczyk, W. M., Katra, M., Szikszay, T. M., Peugh, J., King, C. D., Luedtke, K., & Coghill, R. C. (2023). Spatial Tuning in Nociceptive Processing Is Driven by Attention. *The Journal of Pain*, *24*(6), 1116-1125.
2. Adamczyk WM, Manthey L, Domeier C, Szikszay TM, Luedtke K. Nonlinear increase of pain in distance-based and area-based spatial summation. Pain. 2021a Jun;162(6):1771–80.
3. Adamczyk WM, Szikszay TM, Kung T, Carvalho GF, Luedtke K. Not as “blurred” as expected? Acuity and spatial summation in the pain system. Pain. 2021b Mar;162(3):794–802.
4. Campero M, Bostock H. Unmyelinated afferents in human skin and their responsiveness to low temperature. Neurosci Lett. 2010 Feb;470(3):188–92
5. Compte, A., & Wang, X. J. (2006). Tuning curve shift by attention modulation in cortical neurons: a computational study of its mechanisms. Cerebral Cortex, 16(6), 761-778.
6. Defrin R, Givon R, Raz N, Urca G. Spatial summation and spatial discrimination of pain sensation. Pain. 2006 Dec;126(1):123–31.
7. Defrin R, Sheraizin A, Malichi L, Shachen O. Spatial summation and spatial discrimination of cold pain: Effect of spatial configuration and skin type. Pain. 2011 Dec;152(12):2739–45.
8. Hoeppli, M. E., Nahman-Averbuch, H., Hinkle, W. A., Leon, E., Peugh, J., Lopez-Sola, M., ... & Coghill, R. C. (2022). Dissociation between individual differences in self-reported pain intensity and underlying fMRI brain activation. Nature communications, 13(1), 3569.
9. Julien N, Marchand S. Endogenous pain inhibitory systems activated by spatial summation are opioid-mediated. Neurosci Lett. 2006 Jul;401(3):256–60.
10. Klement W, Arndt JO. The role of nociceptors of cutaneous veins in the mediation of cold pain in man. J Physiol. 1992 Apr 1;449(1):73–83
11. Marchand S, Arsenault P. Spatial summation for pain perception: interaction of inhibitory and excitatory mechanisms. Pain. 2002 Feb;95(3):201–6.
12. Reid, E., Harvie, D., Miegel, R., Spence, C., & Moseley, G. L. (2015). Spatial summation of pain in humans investigated using transcutaneous electrical stimulation. The Journal of Pain, 16(1), 11-18
13. Quevedo AS, Coghill RC. Filling-In, Spatial Summation, and Radiation of Pain: Evidence for a Neural Population Code in the Nociceptive System. J Neurophysiol. 2009 Dec;102(6):3544–53.
14. Quevedo AS, Mørch CD, Andersen OK, Coghill RC. Lateral inhibition during nociceptive processing. Pain. 2017 Jun;158(6):1046–52.
15. Stevens, S. S. (1970). Neural Events and the Psychophysical Law: Power functions like those that govern subjective magnitude show themselves in neurelectric effects. Science, 170(3962), 1043-1050.
16. Streff, A., Kuehl, L. K., Michaux, G., & Anton, F. (2010). Differential physiological effects during tonic painful hand immersion tests using hot and ice water. European journal of pain, 14(3), 266-272.
